# Supplementary material for: Exploring if and how evidence-based practice of occupational and physical therapists evolves over time: A longitudinal mixed methods national study
Source: PLoS One. 2023 Mar 31;18(3):e0283860. doi: 10.1371/journal.pone.0283860 (PMC10065251; doi:10.1371/journal.pone.0283860)
Supplement: S1 Appendix — (DOCX) [file pone.0283860.s001.docx]

**S1 Appendix. COREQ Criteria used for reporting qualitative phase**

*Developed from Tong, A., Sainsbury, P., & Craig, J. (2007). Consolidated criteria for reporting qualitative research (COREQ): A 32-item checklist for interviews and focus groups. International Journal for Quality in Health Care, 19(6), 349–357.*

| **No. Item** | **Guide questions/description** | **Answers** |
| --- | --- | --- |
| **Domain 1: Research team and reﬂexivity** | | |
| *Personal Characteristics* | | |
| 1. Facilitators | Which author/s conducted the focus group? | AT and AR conducted the focus group discussions (FGDs) |
| 2. Credentials | What were the researchers’ credentials? | All authors have masters and/or PhDs in their respective fields. |
| 3. Occupation | What was their occupation at the time of the study? | All researchers are faculty members (associate and full professors) in their respective institutes except MZI (postdoctoral researcher) and MFV (biostatistician). |
| 4. Gender | Was the researcher male or female? | 11 females, 3 males |
| 5. Experience and training | What experience or training did the researcher have? | The team comprised of seasoned researchers with decades of experience in research and established research programs. |
| *Relationship with participants* | | |
| 6. Relationship established | Was a relationship established prior to study commencement? | No known relationship – some co-authors may have taught few as students in entry-level program |
| 7. Participant knowledge of the interviewer | What did the participants know about the researcher? e.g. personal goals, reasons for doing the research | The study background, objectives and FGD details were communicated in advance. All participants received and signed the consent form before the commencement of FGDs. The objective of the study was reiterated at the beginning of FGDs. Both facilitators introduced themselves in the beginning of FGDs to build rapport with participants. |
| 8. Interviewer characteristics | What characteristics were reported about the inter viewer/facilitator? e.g. Bias, assumptions, reasons and interests in the research topic | Both facilitators shared their interests with the participants and explained the study rationale in detail. |

| **Domain 2: study design** | | |
| --- | --- | --- |
| *Theoretical framework* | | |
| 9. Methodological orientation and Theory | What methodological orientation was stated to underpin the study? e.g. grounded theory, discourse analysis, ethnography, phenomenology, content analysis | Content analysis guided by Theoretical Domains Framework. |
| *Participant selection* | | |
| 10. Sampling | How were participants selected? e.g. purposive, convenience, consecutive, snowball | Purposive sampling technique was used. |
| 11. Method of approach | How were participants approached? e.g. face-to-face, telephone, mail, email | We first asked the survey participants to share at the end of survey should they wish to participate in follow up FGDs. Then the volunteers were contacted via emails. |
| 12. Sample size | How many participants were in the study? | Fifteen clinicians (9 OTs and 6 PTs) |
| 13. Non-participation | How many people refused to participate or dropped out? Reasons? | More than half volunteers could not participate because of personal or professional circumstances arising from the pandemic (e.g., illness, redeployment, increased workload etc.) at the time of FGDs. |
| *Setting* | | |
| 14. Setting of data collection | Where was the data collected? e.g. home, clinic, workplace | Online via Zoom communications Inc. |
| 15. Presence of non-participants | Was anyone else present besides the participants and researchers? | No. |
| 16. Description of sample | What are the important characteristics of the sample? e.g. demographic data, date | - All participants were those who also participated in the annual survey. - Participants had to be active practitioners. |
| *Data collection* | | |
| 17. Interview guide | Were questions, prompts, guides provided by the authors? Was it pilot tested? | The guide was not shared with the participants.  No, the guide was not pilot tested, but it was designed and reviewed by 3 TDF experts (AT, AT, AB). |
| 18. Repeat interviews | Were repeat interviews carried out? If yes, how many? | In total, 3 FGDs were conducted; one after each annual survey.  Participants were not repeated in the FGDs. |
| 19. Audio/visual recording | Did the research use audio or visual recording to collect the data? | All FGDs were audio recorded. |
| 20. Field notes | Were ﬁeld notes made during and/or after the interview or focus group? | Field notes were taken but were not used in the data analysis to avoid researcher bias. We used the recordings as the sole source of data, analyzed by independent data analyst. |
| 21. Duration | What was the duration of the interviews or focus group? | 45 to 60 minutes. |
| 22. Data saturation | Was data saturation discussed? | No |
| 23. Transcripts returned | Were transcripts returned to participants for comment and/or correction? | No |
| **Domain 3: analysis and ﬁndings** | | |
| *Data analysis* | | |
| 24. Number of data coders | How many data coders coded the data? | One (TO) and four (MZI, AT, AR, AB) reviewed independently. |
| 25. Description of the coding tree | Did authors provide a description of the coding tree? | No. We performed deductive coding and used TDF domains to develop an understanding of the data. |
| 26. Derivation of themes | Were themes identiﬁed in advance or derived from the data? | We used TDF domains to guide the analysis. |
| 27. Software | What software, if applicable, was used to manage the data? | NVivo (QRS International, Melbourne, Australia) version 12 |
| 28. Participant checking | Did participants provide feedback on the ﬁndings? | No |
| *Reporting* | | |
| 29. Quotations presented | Were participant quotations presented to illustrate the themes/ﬁndings? Was each quotation identiﬁed? e.g. participant number | Yes. Please see Appendix 4. |
| 30. Data and ﬁndings consistent | Was there consistency between the data presented and the ﬁndings? | Yes |
| 31. Clarity of major themes | Were major themes clearly presented in the ﬁndings? | Yes |
| 32. Clarity of minor themes | Is there a description of diverse cases or discussion of minor themes? | Yes |
