# Supplementary material for: Exploring if and how evidence-based practice of occupational and physical therapists evolves over time: A longitudinal mixed methods national study
Source: PLoS One. 2023 Mar 31;18(3):e0283860. doi: 10.1371/journal.pone.0283860 (PMC10065251; doi:10.1371/journal.pone.0283860)
Supplement: S2 Appendix — (DOCX) [file pone.0283860.s002.docx]

**S2 Appendix. Guide for Focus Group Discussions**

Preparation Homework for Participants (to be given 24 hours before)

Think of a patient who was directly under your care and was recently discharged from your caseload(< 7 days ideally or within an appropriate amount of time given your practice setting). Review the details of his/her case, such as what you did when they were under your care, including the decisions that you made (i.e. assessment, treatment) and the clinical process you took as their OT or PT.

**Discussion Script:**

**Preamble:**

**Facilitator**: Have you had a chance to read the consent form? (Yes/ No). Before we get started just a few things to review with you…

**Facilitator**: A quick reminder that this discussion is being audio-recorded and is going to last approximately 45-60 minutes, during which you can stop and take a break at any point that you wish. It doesn’t matter if you say any names of people or places, as all names will be taken out of the transcripts. No confidential or identifying information will appear in any analysis or discussion of the results.

**Facilitator**: Do you have any additional questions for me before we continue?

**Demographic Check-In Questions:**

**Facilitator:** Can you please tell us where you are working right now (workplace setting) and with what population?

Some follow up questions to ask:

Do you work full or part-time?

How many months/years have you been working in this setting?

BEGINNING OF INTERVIEW

**Patient characteristics and some background information**

**1) Facilitator:** Going back to the e-mail with the preparation instructions for today, we had asked you to think about a patient/client you recently discharged within the past week, for the next few minutes, I would like you to share with me some background information about the case.

- Who is the patient and what is the main issue
- What were some of the problems that stood out for you.
  - For example, what was their particular situation?

Some prompts (“tell me about….”):

What was he/her referred to you for?

What was the presenting diagnosis?

What was the mechanism of injury?

What was their past medical and psycho-social history?

**Use of conceptual/practice models/theories/protocols and how these are used**

**1) Facilitator**: Can you share with me if you drew from/used any conceptual/theoretical/practice models when you worked with this client/patient?

*Wait until they answer spontaneously before providing the prompt.

*Prompt:* For example, a model could be chronic pain model, bio-psychosocial model (PT references) *Prompt*: For example, a model could be the Model of Human Occupation (MOHO), or the Canadian Model of Occupational Performance and Engagement (CMOP-E) (OT references)

**2) Facilitator:** Tell me about how you used this model for this client/patient?

*Prompt*: How did the use of the model influence your decision?

**Assessment: what was assessed, how you assessed it**

**1) Facilitator**: Can you tell me about what you assessed with the client/patient? For each thing assessed, tell me how.

**2) Facilitator**: Tell me about which assessment tools you used with this patient/client for each thing you assessed?

*Prompt*: Can you share with me if this was done as a test, using a questionnaire, observation or standardized/non-standardized format?

*If they mention assessing range of motion, muscle strength or even ADLs, it is important to ask, how they conducted these assessments (e.g., did they use observation alone, did they use a goniometer) but do not provide these examples as cues, they are only for the interviewer.

*Prompt:* If a homemade tool was used, please inquire as to how was it developed?

**3) Facilitator**: Tell me about how you decided which algorithms (PT), clinical guidelines or even protocols to use?

**4) Facilitator**: How did you decide to use this specific method for conducting your assessment?

*If the participants do not spontaneously reference experiential/tacit knowledge, scientific knowledge (research evidence), and patient preferences supporting their use of an assessment(s), then the interviewer should prompt them.

*Prompt:* Was it based on any research, or your experience or your patient’s input?

**Treatment: what interventions (goals, means and methods) were used**

**1) Facilitator:** Tell me about what were the treatment/intervention goals?

**2) Facilitator:** For each one, tell me about which interventions/treatments (schemes, approaches) were used?

**3) Facilitator:** Tell me about how you decided to use that intervention(s)? or on what basis you made this decision?

***If the participants do not spontaneously mention anything related to the evidence (i.e. they selected a particular intervention because it was supported by evidence), then they should be prompted, by asking:

*Prompt:* It is important that we understand exactly why an intervention was selected; what factors influenced your decisions/tx plan?

**Termination of process of care: criteria used to determine the end of services (discharge)**

*Prompt: Can you give me an example for this?*

**1) Facilitator**: Describe the discharge process for this patient/client?

*Prompt:* How did you know it was time for your role to come to an end with this patient/client?

**2) Facilitator**: If you could sum it up in a few sentences, tell me about how in an ideal world how you would want the discharge process to be?

**Closing Question:**

**3) Facilitator:** Is there anything else that you would like to add, or elaborate on reflecting on everything that we discussed today about this patient/client and what you did as their OT or PT?

------------------------------------------------------------------------------------------------------------------------

**End of interview:**

Thank the participants for volunteering their time to speak to you today, and that they can learn more about the study by looking at our website (https://www.mcgill.ca/keep-lab/projects) or by following us on Twitter/Instagram (@KEEPlab, #bigOTPTproject, keep_lab)
