# Supplementary material for: Exploring if and how evidence-based practice of occupational and physical therapists evolves over time: A longitudinal mixed methods national study
Source: PLoS One. 2023 Mar 31;18(3):e0283860. doi: 10.1371/journal.pone.0283860 (PMC10065251; doi:10.1371/journal.pone.0283860)
Supplement: S3 Appendix — (DOCX) [file pone.0283860.s003.docx]

**S3 Appendix. Time points wise content analysis guided by Theoretical Domain Framework (TDF)**

| TDF Domains | Questions used in FGD (n) | Time point | Utterances (n) | Specific beliefs  (n) | Facilitators  n (%) | Barriers  n (%) | No influence  n (%) |
| --- | --- | --- | --- | --- | --- | --- | --- |
| Knowledge | 2 | T1 | 23 | 11 | 12 (52.2) | 6 (26.1) | 5 (21.7) |
|  |  | T2 | 38 | 17 | 25 (65.7) | 0(0) | 13 (34.2) |
|  |  | T3 | 18 | 10 | 17 (94.4) | 1 (5.5) | 0 (0) |
| Beliefs about capabilities | 2 | T1 | 9 | 4 | 5 (55.5) | 3 (33.3) | 1 (11.1) |
|  |  | T2 | 11 | 1 | 7 (63.3) | 4 (36.6) | 0 (0) |
|  |  | T3 | 16 | 9 | 12 (75.0) | 4 (15.0) | 0 (0) |
| Behavioral regulation | 3 | T1 | 22 | 6 | 20 (90.9) | 2 (9.1) | 0 (0) |
|  |  | T2 | 24 | 8 | 17 (70.8) | 4 (16.6) | 3 (12.5) |
|  |  | T3 | 12 | 12 | 11 (91.6) | 1 (8.3) | 0 (0) |
| Skills | 1 | T1 | 6 | 4 | 3 (50.0) | 1 (16.6) | 2 (33.3) |
|  |  | T2 | 17 | 8 | 16 (94.1) | 0 (0) | 1 (5.9) |
|  |  | T3 | 11 | 5 | 11 (100) | 0 (0) | 0 (0) |
| Beliefs about consequences | 3 | T1 | 8 | 4 | 5 (62.5) | 3 (37.5) | 0 (0) |
|  |  | T2 | 19 | 7 | 16 (84.2) | 3 (15.7) | 0 (0) |
|  |  | T3 | 6 | 5 | 3 (50.0) | 3 (50.0) | 0 (0) |
| Environmental context and resources | 2 | T1 | 13 | 6 | 5 (38.4) | 8 (61.5) | 0 (0) |
|  |  | T2 | 58 | 10 | 27 (46.5) | 30 (51.7) | 1 (1.7) |
|  |  | T3 | 30 | 10 | 10 (33.3) | 17 (56.6) | 3 (10.0) |
| Social influences | 1 | T1 | 9 | 3 | 4 (44.5) | 5 (55.5) | 0 (0) |
|  |  | T2 | 14 | 4 | 10 (71.4) | 2 (14.2) | 2 (14.2) |
|  |  | T3 | 11 | 5 | 6 (54.5) | 5 (45.4) | 0 (0) |
| Social/ professional role and identity | 1 | T1 | 8 | 2 | 7 (87.5) | 1 (12.5) | 0 (0) |
|  |  | T2 | 7 | 3 | 7 (100) | 0 (0) | 0 (0) |
|  |  | T3 | 9 | 4 | 9 (100) | 0 (0) | 0 (0) |
| Emotions | 2 | T1 | 7 | 4 | 0 (0) | 7 (100) | 0 (0) |
|  |  | T2 | 15 | 5 | 7 (46.6) | 7 (46.6) | 1 (6.6) |
|  |  | T3 | 9 | 8 | 3 (33.3) | 5 (55.5) | 1 (11.1) |
| Goals | 2 | T1 | 6 | 3 | 3 (50.0) | 1 (16.6) | 2 (33.3) |
|  |  | T2 | 3 | 3 | 3 (100) | 0 (0) | 0 (0) |
|  |  | T3 | 8 | 7 | 6 (75.0) | 1 (12.5) | 1 (12.5) |
| Decision processes | 2 | T1 | 8 | 4 | 4 (50) | 4 (50) | 0 (0) |
|  |  | T2 | 8 | 3 | 2 (25) | 1 (12.5) | 5 (62.5) |
|  |  | T3 | 0 | 0 | - | - | - |
| Reinforcement | 1 | T1 | 5 | 3 | 5 (100) | 0 (0) | 0 (0) |
|  |  | T2 | 21 | 11 | 19 (90.5) | 0 (0) | 2 (10.5) |
|  |  | T3 | 6 | 4 | 6 (100) | 0 (0) | 0 (0) |
| Optimism | 1 | T1 | 5 | 1 | 5 (100) | 0 (0) | 0 (0) |
|  |  | T2 | 0 | 0 | - | - | - |
|  |  | T3 | 0 | 0 | - | - | - |
| Intention | 1 | T1 | 5 | 5 | 4 (80) | 0 (0) | 1 (20) |
|  |  | T2 | 3 | 3 | 3 (100) | 0 (0) | 0 (0) |
|  |  | T3 | 11 | 11 | 11 (100) | 0 (0) | 0 (0) |
| Total | 24 |  | 519 | 233 | 348 (67.05) | 127 (24.47) | 44 (8.47) |

- Facilitators represent the number and percentage of utterances perceived to increase the use of EBP.
- Barriers represent the number and percentage of utterances perceived to reduce the use of EBP.
- No influence represents the number and percentage of neutral utterances that were neither perceived as facilitators nor barriers to the use of EBP.
