# Supplementary material for: Exploring if and how evidence-based practice of occupational and physical therapists evolves over time: A longitudinal mixed methods national study
Source: PLoS One. 2023 Mar 31;18(3):e0283860. doi: 10.1371/journal.pone.0283860 (PMC10065251; doi:10.1371/journal.pone.0283860)
Supplement: S4 Appendix — (DOCX) [file pone.0283860.s004.docx]

**S4 Appendix. Summary of OTs and PTs specific beliefs and sample quotes assigned to the relevant domains**

| **TDF Domain** | **Time point** | **Specific beliefs (number of utterances)** | **Salient quotes** |
| --- | --- | --- | --- |
| **Knowledge** | T1 | *EBP – meaning (n=13):*   - EBP is context & client dependent (n=5) - EBP is consulting literature & other health-care professionals (n=4) - EBP is just consulting literature and ensuring practice is based on evidence (n=2) - EBP can involve consulting patients, specifically in less researched fields (n=1) - EBP is facilitated by the understanding research methods (n=1)   *EBP – sources (n=8):*   - Articles, meta-analyses, reviews (n=3) - Online research of abstracts (n=2) - Gold-standards for assessments (n=1) - Vetted online resources (n=1) - Research projects (n=1) - Online knowledge about existing EBP sources (n=2) | *“So, I guess for me it is a balance of consulting the literature and maybe some other professional OTs to get advice, and then applying it to my environment.”*  *“I tend to go straight to journal articles, hopefully review and meta-analyses articles. But there's not really an engine equivalent to Stroke Engine for school health, so I'm pretty limited. But I do often consult journals.”*  *“I've attended a few professional development opportunities, and I love them. For example, I attended one on social thinking. But then actually applying it to my practice just seems a bit difficult. It would take a lot of time, and I would have to prioritize it and not do other things in order to make it happen in my practice.”* |
|  | T2 | *EBP – meaning (n=14):*   - EBP refers to consulting literature and ensuring practice is based on evidence (n=4) - EBP is context & client dependent (n=1) - EBP is facilitated by the understanding research methods (n=1) - EBP refers to using standardized assessment and effective interventions (n=4) - EBP can be supported by clinical evidence (n=2) - EBP provides a framework (n=1) - EBP becomes less relevant with experience (n=1)   *EBP – sources (n=24):*   - Vetted online resources (n=4) - Reading articles, meta-analyses, reviews (n=2) - Databases (n=6) - Colleagues (n=4) - Continued education and training that provide relevant references (n=2) - University courses’ notes (n=2) - Theoretical models (n=1) - Social media (n=1) - Sources may vary depending on level of experience/starting to work in a new setting (n=1) - In-services within department, inter-department, inter-institutional (n=1) | *“I think my biggest difference is the fact that I'm using more anecdotal evidence, like clinical evidence, from what I've seen in practice rather than just what the literature has said, only because I'm finding that a lot of what's being recommended within the literature may be applicable to some populations but not all of them, and so knowing how to apply that evidence and what works for what population has been more of what I rely on rather than the literature right now.”* (P2)  *“So, I would say overall my evidence-based practice has become less formal than it would have been as a student.”* (P3)  *“I rely a lot on what other people have found too, and I probably shouldn't, but I do take it at face value and kind of go, “Oh, well they said that and they are quite senior, and they seem to know what they're doing, so I'm just going to rely on that.”* (P1)  *“Other information sources on the internet, I mean social media, are a little bit easier to access, and a little more time-efficient”* (P3) |
|  | T3 | *EBP – meaning (n=13):*   - EBP is useful in guiding a problem-solving approach in clinical practice (n=1) - EBP is useful in managing complex patients with comorbidities (n=2) - EBP is useful in standardizing approaches with new populations (n=1) - EBP has become more focused with experience (n=3) - EBP is used less because of lack of evidence that is population specific (n=1) - EBP refers to consulting literature and ensuring practice is based on evidence (n=2) - EBP has changed to become evidence informed practice or triangulating between patients’ perspectives, learned material and new evidence from literature (n=2) - EBP refers to continue learning and being up to date with the evidence (n=1)   *EBP – sources (n=2):*   - Online resources that are easily accessible and webinars (n=1) - Subscriptions to journals (n=1) | *“Where it (EBP) becomes more useful for me is when I’m starting to sort of push the boundaries of my scope a little bit and sort of starting to see our patients are very complex and I’m a little less well versed in that in terms of my clinical experience so then I’m sometimes looking more to research articles to try to beef up that part of my practice.”* (P1)  *“I think what is a little bit different than before is that I’m not going to literature for one thing only. Now I sort of look at a patient and see where are my learning gaps and how can I fill them, as opposed to before it was ‘Oh I’m learning about this new topic, I’m just going to try to go at everything and try to narrow it down’.”* (P4) |
| **Beliefs about capacities** | T1 | Self-confidence is important to be an EB practitioner (n=3)  Facilitators:   - Group discussions (n=2) - Being up to date with EBP (n=1)   Barriers:   - Senior clinicians & being early in their career path (n=3) | *“Well, at that point I was a student. So, I used to smile and nod along and hoped I passed, to be honest. In my current practice, though, when they [seniors] give me advice that I don't think is evidence-based, I ignore it. I don't go out of my way to correct them. I have just started [practicing], so I think like, “Um, that's not evidence-based, or I don't think it's evidence-based”. but I'm kind of new and haven’t really done all the work that they've done, so you know, it's kind of difficult to be the lowest tier and to tell people that they're wrong.”* (P1) |
|  | T2 | Self-confidence is important to be an EB practitioner (n=3) | *“I have an example from private practice in Physio. So, it was a middle-aged man who came in for intense back pain and based on all the research that I have read, back pain self-resolves within a couple weeks, could be a couple of months, but usually it can self-resolve, and it's usually not anything serious. But with that research in mind it helped me inform on how to approach the situation, because he was obviously very concerned and very distraught that, “What if I have a disc herniation?” or “What if I can't walk again?” so it [evidence] helped me to stay focused and objective about how to deal with his back pain, and in the end it worked out well. He returned back to his normal life. But I think without reading the evidence I might have considered imaging or something like that, but it wasn't really necessary. I think it [EBP] gave me a lot of confidence to actually deliver the best treatment, and it just helped to make the overall experience of physiotherapy much better.”* (P1) |
|  | T3 | Self-confidence is important to be an EB practitioner (n=3)  Facilitators:   - Ample evidence for certain population groups (n=2) - Becoming more efficient in search and understanding evidence (n=2) - Learning how to question practice and find solutions (n=1) - Having more specific research questions when searching for evidence (n=1)   Barriers:   - Lack of evidence for certain population groups (n=2) - Senior clinicians (n=2) - Difficulty generating specific research questions for certain population groups (n=1) - Lack of intrinsic motivation (n=3) | *“Now it [EBP] is very clinical based. Now I know what I need from evidence and how do I find it. So, I spend way less time and I’m way quicker to go through “OK this is helpful, this isn’t… let me read the summary, take what I need and go with it”. So I’m not spending too much time because now I know what I’m looking for.”* (P1) |
| **Behavioral regulation** | T1 | - Strategies to stay up to date with EBP (n=14): - Being part of a group to receive information, resources, webinars, and links to continuing education (n=5) - Allocating time to review evidence (1) - Consulting colleagues for resources (n=4) - Consulting social media (n=3) - Consulting literature & online resources (n=1) | *”I’m on a couple mailing lists for Permobil. So, it gives me an idea about which latest products are coming out for seating and for communication devices. It's a little bit biased because it's coming from the company, but it at least gives me sort of a heads up that this item is available now, and I can look into it.”* |
|  | T2 | - Strategies to stay up to date with EBP (n=13): - Consulting colleagues for resources (n=5) - Being part of a group to receive information, resources, webinars, and links to continuing education (n=3) - Consulting social media (n=2) - Consulting literature & online resources (n=1) - Email alerts/newsletters on new evidence (n=2) | *“I have a newsletter, well it's like an email alert, for new articles that pop up in my areas of interest. I find it very helpful for my EBP.”* |
|  | T3 | - Strategies to stay up to date with EBP (n=3): - Consulting colleagues for resources (n=1) - Consulting literature & online resources (n=2) - EBP can be time and resource consuming (n=2) | *“I’ve used it [EBP] for patient education or client education in the past. I’ve had a couple clients with back injuries. It can be very challenging if the clients aren’t buying into the fact that exercise is really one of the best ways to heal back sprains. So in the past, I have had to dig up some research for clients to kind of show them that active rehabilitation is one of the best ways to help healing the back sprain and that we are not just pushing this treatment option.”* |
| **Skills** | T1 | - Critical appraisal of literature (n=4) - Level of experience (n =2) - Self-assessment, self-awareness (n=2) - Communication abilities (n=1) - Time management skills (n=1) | *“I think there's a self-awareness component where you have to be willing to examine what you're doing and why you're doing it.”* (PB)  *“I think time management skills are really important. Because for me it is the biggest challenge. I just don't have the free time. Also, I think confidence in what I'm doing and what I'm able to do is important too.”* (PC) |
|  | T2 | - Critical appraisal of literature (n=3) - Level of experience (n =3) - Self-assessment, self-awareness (n=1) - Communication abilities (n=3) - Setting priorities – being proactive – self-regulation (n=4) - Improved clinical reasoning (n=2) - Educating client on EBP (n=1) - Subjective examination (n=1) - Interprofessional skills with colleagues (n=1) | *So, in EBP, there are different pillars. There's the literature, there's your clinical expertise, and then the client values. I would say that the clinical reasoning pillar for me has grown. Now I actually take little evidence straight from the literature.”* |
|  | T3 | - Ability to searching databases and understanding research (n=6) - Ability to narrow down the search (n=2) - Recognize that there is a need for learning (n=1) - Self-assessment, self-awareness (n=1) - Management skills (n=1) | *“I think what’s helpful to EBP is being able to narrow down your search and knowing what keywords you should use to pull up the abstracts. Have a bit of a look at it through a critical lens and understand ‘does it apply to my patient or not? If it does, is it worth my time to then find access to this journal and go through it?’ And then apply it.”* |
| **Beliefs about consequences** | T1 | - Adapting EBP to contexts can be challenging (n=3) - Effectiveness/better outcomes (n=2) - Professional recognition (n=2) - Harm reduction (n=1) | *It's a lot of juggling and a lot of trying. To balance best evidence purely scientifically with the lived experience of my clients and their wishes is sometimes difficult. They [patients] are adults and they're cognitively capable, and so it's just like a constant negotiation that can be difficult.”* (P?) |
|  | T2 | - Effectiveness/better outcomes (n=4) - Adapting EBP to contexts can be challenging (n=3) - Professional recognition (n=3) - Harm reduction (n=2) - Credibility (specifically in complex cases & situations) (n=3) - Legalities (n=3) - EBP use to objectively measure response to treatment (n=1) | *“…without evidence we're just really shooting in the dark right? So, having that science-based approach I think is what makes physio and occupational therapy, and all other professions what it is right now.”* (P1)  *“I think EBP ensures that we're not causing harm when we're working with people, as OT's quite a broad profession and you could be working in so many different areas, and there are so many ways to cause harm with your clients, so I think following concrete information that we do have to make sure that we're not going to be messing up the people we're working with is... also just kind of cover your back basically.”* (P3) |
|  | T3 | - Adapting EBP to contexts can be challenging (n=2) - Credibility (specifically in complex cases & situations) (n=1) - Effectiveness/better outcomes (n=1) - EBP increases patients’ understanding and improves clinician-patient communication (n=1) - Conflicting evidence or low-quality evidence (n=1) | *“I think it [EBP] is difficult because these days there are research articles to support any point of view… you could find an article that says one thing works and find another that says it doesn’t work, or another that says it only works under certain conditions… so it’s very difficult because you really have to be critical of the studies and their methodologies.”* (P2)  *“One thing I would say that’s an advantage of EBP is knowing that I am providing the most current research-based practice to my clients and educating them that this is the best level of evidence and explaining the rational of treatment helps them to understand why we develop treatment protocols, plans, based on their goals and why we are doing certain things. So, it [evidence] helps me to explain it to the patients and it also gives me the confidence.”* (P4) |
| **Environmental context and resources** | T1 | - Time constraint - lack of dedicated or protected time (n=5) - Access to search databases (n=3) - Organized topic discussions (n=2) - Organizational culture (n=1) - Access to resources & products (n=1) - Lack of support (n=1) | *“Treatment for me has been quite a tricky thing to balance with evidence, and with what's realistic in my practice, and with my caseload. So, for treatment I've been trying to do some readings on studies, but there's a big-time constraint for me.”* (P?) |
|  | T2 | - Organizational culture (n=14) - Time constraint - lack of dedicated or protected time (n=13) - Access to search databases & librarian support (n=10) - Access to resources & products (n=2) - Support from colleagues (lack vs. presence) (n=1) - Courses/continued education (n=7) - Financial supports (n=5) - New setting and new population (adaptation) (n=3) - Clinician-researcher (n=2) - Physical space (n=1) | *“As a student, things are a little bit more accessible in terms of having access to all of these databases with research articles. People don't always have that when they're not a student, so it makes it a little bit more challenging to access if I was interested in formulating a specific research question. So, that's kind of a limitation.”* (P2)  *“We have a common area of interest that we all tend to sit and chart, so it's nice to have people to bounce ideas off of, and I'm very fortunate that I work in a clinic with a lot of very smart people who have access to firsthand evidence. So, it's nice to be able to just sit around, kind of chat through some problems.”* (P3) |
|  | T3 | - Access to search databases & librarian support (n=9) - Time constraint - lack of dedicated or protected time (n=8) - Affiliation to a teaching institution (n=2) - Evidence availability (or lack of thereof) (n=2) - Conflicting or low-quality evidence (n=2) - Courses/continued education (n=2) - Organizational culture (n=2) - Subscriptions to journals and online materials (n=1) - Organized topic discussion (n=1) - Access to resources and products (n=1) | *“I would say funding-wise they [workplace] support me at least quite well, but it's very much self-directed in terms of finding the right CPD activities. They don't really give us direction of where to go, or which courses we should take, but they're kind of like, “Here's this money, and here are these days off you can use to do whatever interests you.” But it's usually very much self-directed.”* (P6) |
| **Social influences** | T1 | - Colleagues’ perspectives/colleagues as resources (n=5) - Client's expectations & previous experiences (n=3) - Group conformity (n=1) | *“I think for me it's going to be the lawyers, insurance providers and treatment funding companies that can play a role in EBP.”* (P1) |
|  | T2 | - Colleagues’ perspectives/colleagues as resources (n=7) - Client's expectations & previous experiences (n=4) - Group conformity (n=1) - Institution (lawyers, insurance companies, funding partners, upper management) (n=2) | *“I find that only a small subset of clients will ask for the evidence behind treatments, but most clients that I've had so far just want to get better, however method that you want to go about. So, it's more about the institution who cares a lot about the evidence of what I'm doing.”* (PC?) |
|  | T3 | - Colleagues’ perspectives/colleagues as resources (n=4) - Client's expectations & previous experiences (n=4) - Institution mandate with respect to EBP and research (n=1) - Institution that is not affiliated to a teaching unit or teaching hospital (n=1) - Colleagues’ perspectives (n=1) | *“At our hospital, they [administration] always say that evidence and research is a huge part of the institute, and they dedicate so much time and money into research itself. They want us to be evidence-based practitioners. So admin role is very important to EBP, I think.”* (P3) |
| **Social/ professional role and identity** | T1 | - Commitment to provide EBP to patients (n=5) - Commitment to the profession (n=3) | *“Yeah, I think we definitely have a responsibility towards our clients to be evidence-based. And for me, I might be the only resource that some of these families have. That puts even more weight on me to do something that will be effective and helpful for them.”* (PC?) |
|  | T2 | - Commitment to the profession (n=5) - Commitment to provide EBP to patients (n=1) - Clinician-researcher role (n=1) | *“During the 2 years that I'm practicing, the priority of getting my clients better has been number one, and that has been a resounding goal that I have set for myself. So, whatever gets the client better quicker and faster works for me.”* (P2) |
|  | T3 | - Becoming a proactive EB practitioner with experience where EBP is deeply embedded in the professional role identity (n=4) - EBP is part of training culture (n=2) - Commitment to the profession (n=2) - Clinician researcher (n=1) | *“I think it's just the legitimacy of the profession. As physio, we've come a long way but there is still, from a societal perspective sometimes, especially in older populations, there are stereotype beliefs which are still very prevalent. So, saying ‘Yes, this is legitimate thing. Here is the proof of how it works’ can really make patients understand what we do and why.”* (P3) |
| **Emotions** | T1 | - Frustrations with mismatch between EBP and client's preferences (n=2) - Anxiety (n=2) - Feeling uneasy with respect to client's safety (n=2) - Frustrations with mismatch between EBP and team's perspectives (n=1) | *“It can feel sometimes like there is conflict. I had a patient ask me yesterday for a weighted vest, which might be risky and might not have great evidence, but they hear about it and they think it's legitimate. Then saying no to the patient kind of feel like you're not being client-centered.”* (PA) |
|  | T2 | - Feeling reassured – stress attenuation (n=6) - Guilt (n=4) - Frustration regarding quick changes in EBP (n=2) - Boredom vs. excitement (n=1) - Feeling overwhelmed (n=1) | *“It happened fairly recently where I saw a client. I came up with a plan in my head and then went back to the literature because I was like, “Am I missing anything? Am I doing everything that I need to?” My plan that I had made in my head, which obviously is informed by my training, was on point, and I was like, “Oh! Good work for me.” (Laughs) It was exciting to know that my plan was absolutely according to the literature.”* (P?)  *“I sometimes feel guilty because one moment I read research and it's like, “Oh this is the way to go,” and then I do it and then the next day it's like, “Oh, you shouldn't do this,” and now it's like, “Oh man, I feel like a bad physio.””* (P1) |
|  | T3 | - Excitement and feeling grateful (n=1) - Feeling empowered (n=2) - Feeling of comfort (n=1) - Feeling of a making a contribution to patients’ outcomes and colleagues’ knowledge (n=1) - Frustrations with not findings exactly what you are looking for (n=1) - Feeling overwhelmed (n=1) - Feeling intimidated, daunting (n=1) - Feeling like a student or a trainee again (n=1) | *“What I also find really frustrating is that the evidence changes, and once I get comfortable with something and I've done it so many times, and then the following year there's evidence that says, “Oh, this does absolutely nothing, why have you been doing this?” That's been really frustrating because you have to keep yourself on top of things.”* (P3)  *“I think sometimes it’s exciting that there is something new to know and maybe bring it back to help my patient and then if it does work, it is very gratifying.”* (P4) |
| **Goals** | T1 | - Types of EBP goals (n=3) - Intention to set goals related to EBP (n=1) - Intrinsic motivation or lack thereof (n=2) | *“I think for me I would want to start small right now, because I'm just feeling very busy. So, even to read 1 article a month would be a baby step goal. And I would like to apply it somehow into my practice. I have yet to actually do it, but maybe I need to write it down, maybe I need to have it at my office. I think it could be a really good and helpful strategy.”* (PC?) |
|  | T2 | - EBP goals matching population served (n=1) - Participate more to online learning (n=1) - Increase knowledge in EBP (n=1) | *“So, one of my goals is to do a little bit more online-based learning. I have a family now, so kind of time is an issue, and this is just a way for me to stay a little bit more up-to-date. I will have to fit it in on my work time because doing it at my own time doesn't always work with my family.”* (P2)  *“I would want to set my goals according to the type of clients that I see in my current job. Like, when I first started, I was doing mostly seeing pediatric clients, so my goals around evidence-based practice were around pediatrics, and now I'm seeing more adult mental health clients, so my goals have shifted to make sure that I'm adequately addressing the needs of the clients that I'm seeing most often now.”* (P?) |
|  | T3 | - To stay up to date with the EBP (n=2) - To become more efficient in understanding research and seeking high quality evidence (n=1) - Expectations not meeting reality (n=1) - To have sustainable checkpoints of self-assessment for EBP practice (n=1) - To incorporate more EBP in current clinical practice (n=1) - To set realistic expectations with respect to EBP (n=1) - EBP goals matching population served (n=1) | *“I think when I first graduated, I didn’t really make any goals but in the past two years, I did make a goal in that if there was a certain area or condition that I wasn’t too sure how to treat I made an effort to do evidence-based practice, I find that it helps to guide my treatment and kind of give me a direction. Moving forward, as I said, I would like to incorporate even more evidence into my practice in the future.”* (P2) |
| **Decision processes** | T1 | - Decision is based on balanced use of EBP and clinical reasoning (n=4) - Decision is based on harm-reduction & safety (n=2) - Reflection (n=1) - Test the waters (n=1) | *“I think, I've had to do a lot of wrestling with the best evidence, my personal choice involved with my population, and how my patients choose to live the rest of their lives. So I think that's maybe an area where I struggle a bit.”* (P2) |
|  | T2 | - Decision is based on balanced use of EBP and clinical reasoning (n=4) - Decision is based on harm-reduction & safety (n=1) - Priority setting (reviewing evidence vs. clinical work) (n=3) | *“A big use of evidence for me is harm-reduction. Sometimes I have unique client situation where they are doing things that may be harmful, and they're willing to take bigger risks just to become well and functional. So, for me a lot of times I ask myself that whatever question they're asking me, is it going to cause harm if I'm wrong.”* (PB) |
|  | T3 | - - | - |
| **Reinforcement** | T1 | - Patients', families', and other professionals' positive feedback (n=3) - Personal sense of integrity & satisfaction (n=1) - Incentive to make different recommendations (n=1) | *“Yeah, I think a big reward for me is when families or teachers come to me and say “that was so helpful, I've seen huge gains in this student”, I find that super rewarding. And then it helps me kind of target, okay, that's the treatment we did, maybe we should keep doing it because it worked so well.”* (PC)  *“I think the reward is always just clients getting better or improving in whatever way you're treating them, especially when it’s happening as predicted in the literature, or better. Sometimes you try something new, or slightly different than what you typically do, and you're not sure how it's going to work, and then if you get a really drastic improvement of what's expected, and I think that's the most rewarding outcome, or reason for using evidence-based practice.”* (P2) |
|  | T2 | - Patients', families', and other professionals' positive feedback (n=6) - Personal sense of integrity & satisfaction (n=1) - Measure and compensation (n=3) - Dedicated/protected time for EBP (n=2) - EBP that is packaged right (n=2) - Financial aspect (n=2) - Becoming an expert in the area and share knowledge with others – mentorship (n=1) - Bonuses (n=1) - EBP integrated into interview/performance review of the clinician (n=1) - Providing strong justification for 3^rd^ party (n=1) - Supportive employer (n=1) | *“I mean, the financial aspect is certainly one, but just the ease of access. I think in today's society where attention span is only 10 seconds, I think having research presented as attractive, or as attention-grabbing as other competing things is definitely a huge incentive to actually consume more evidence-based articles.”* (P1) |
|  | T3 | - Personal sense of integrity & satisfaction (n=3) - Increase in knowledge (n=1) - Bonuses (n=1) - Patients', families', and other professionals' positive feedback (n=1) | *“I think for me, it’s just knowing that I’m doing the best that I can and providing the highest level of care to my clients, and educating them on that because when they are well informed then they feel like wow, I’m really getting the top care that I can… and if I can’t, then I explain that to them and where they could go afterwards, once they are done in-patient. So I think it’s just again, that comfort in knowing that I’m doing everything that I can and being the best clinician that I can be.”* (P3) |
| **Optimism** | T1 | - Overcoming barriers (n=5) | *“Yeah, I would agree. I try to allocate maybe few hours every Friday, but probably only happens like once a month to consult the literature, and part of that is guilt of feeling like I have to be elsewhere, whether it's in a school, or answering my emails, so that I would say is the main reason why I don't spend more time in my work hours, and then the odd weekend I will bring something home to read, like this weekend I'm reading about constraint-induced movement therapy, because I was asked to maybe help with that in the school. So, a bit of both; in work and outside of work.”* (PC) |
|  | T2 | *-* | - |
|  | T3 | *-* | - |
| **Intention** | T1 | - Incentive (n=3) - Being knowledgeable about practices (n=1) - Evidence is providing grounds for your practice (n=1) | *“For me, I think it's definitely the clients. I just can't think of any other reason besides benefiting the people who I'm working for, and the families, and the children, and feeling like I can help, and have an impact on their quality of life.”* (PC)  *“There's this idea of like, “oh, we will provide some kind of mysterious therapy and it will improve outcomes”, but it does not because it's not real. To actually go to the evidence and look at data, and to have that grounding is really personally satisfying for me, because I just like to live in reality.”* (PA) |
|  | T2 | - Incentive (n=1) - Driven by improving patient’s outcomes & providing best care (n=2) | *“I think success for patients is my biggest motivator. So, my goals usually surround, they're really short-term, and if they involve evidence it's “I need to get better at this”, and it needs to be between now and the next time I see the patient. It's entirely based on how the patient's feeling, and if they're getting better.”* (P2) |
|  | T3 | - Learning institution with emphasis on EBP and/or problem solving through which the passion for EBP develops (n=3) - Driven by improving patient’s outcomes & providing best care (n=3) - Developed sense of curiosity (n=2) - Incentives from employer (n=1) - Not wasting time on things that do not work (n=1) - Being knowledgeable about practices (n=1) | *“Yeah, I usually will seek the evidence when the client is not getting better, something's taking longer than usual, or it's just something that I don't know yet, that's when I will usually seek out the evidence.”* (P4) |

NOTE: Beliefs are listed in descending order (from high to low frequency).
