# Supplementary material for: Exploring if and how evidence-based practice of occupational and physical therapists evolves over time: A longitudinal mixed methods national study
Source: PLoS One. 2023 Mar 31;18(3):e0283860. doi: 10.1371/journal.pone.0283860 (PMC10065251; doi:10.1371/journal.pone.0283860)
Supplement: Attachment — Submitted filename: Review EBP MM.docx [file pone.0283860.s005.docx]

Review

Thank you for the opportunity to review the manuscript entitled "Exploring how evidence-based practice of occupational and physical therapists evolves…" The manuscript makes a potentially important contribution to the body of literature on use of EBP by allied health professionals, by reporting a study that investigated use of EBP and its evolution over the first three years of practice after graduation among Canadian physiotherapists and occupational therapists, and what factors influenced the use. The study combined quantitative and qualitative methods. The paper is generally well written and the study seems well conducted and employs an ambitious methodological approach analysing two different types of data collected at different time points. It presents original research and, as far as I can tell, meets PlosOne’s publication criteria.

Below are my comments that I hope will improve consistency, conciseness and clarity of the paper, after which I think the paper could be accepted for publication.

**Abstract**

-I recommend fewer abbreviations in the abstract. For example, EB, FGD, GBTM could be written out (the sentence in Results that begins with GBTM could be reworded). Abbreviations in the beginning of a sentence is against most writing guidelines.

-The study aim is not formulated the same as in the main text.

-Please state the response rate for the survey. Maybe also the retention rates.

**Background**

-Could you provide more examples of EB activities? Simply ”informal information sharing…” seems quite limited, surely there must be other examples of other types of activities?

-Please avoid using abbreviations in the beginning of a sentence (EB, but other abbreviations elsewhere in the manuscript).

-Not all references 6-9 seem to deal with EBP in rehabilitation services. Please review and, if appropriate, select more relevant papers to support the statement.

-The paragraph on the top half of page 6 could be written more concisely. For example, author names do not need to be stated; what the study did does not need to be stated, only their findings relative to the point you wish to make. Several of the cited papers say more or less the same so could be merged together.

**Methods**

*Study design and setting*

-Although this heading says so, the study setting is not described here.

-Please state what reporting guidelines you’ve used (e.g. STROBE, COREQ), and make sure you’ve followed them in your reporting. There are currently a few aspects, particularly in the Procedure sections that would benefit from your providing more details. This would certainly also increase transparency and credibility of the work and findings, especially the qualitative part.

-Please elaborate on the chosen type of Mixed methods and describe your integration strategies, i.e., how and where in the study process you’ve integrated the quantitative and qualitative data, whether the two data types were equally weighted/prioritised, etc. Consider moving the first three lines under Phase 2, qualitative to the design section, possibly also adding a line about Phase 1 as an introduction to the phase 2 part. This is important to improve clarity concerning your study design and type of Mixed methods used.

-Please add ”explanatory” before sequential, as you have done in the abstract, and in line with the classification typology of MM designs.

-Please consider whether you could present the results in a more integrated manner. Presently, both analyses and results are presented as separate entities rather than integrated - so what does the integration consist of? This could be clarified.

*Procedure*

-The section would benefit from some more details. For example, where were the FGDs held, how long were the sessions, who moderated, etc. Please follow a reporting guideline for qualitative studies, e.g., COREQ (all items may not be applicable). Please consider providing the discussion guide in an appendix (see below regarding terminology).
-Please consider presenting the 14 TDF domains, in text, table or figure.
-Please add the original reference for the TDF (Michie 2005). (I have no relation to the author).

*Data analysis*

-There is no mention of the analytical method/approach used. Content analysis is mentioned in Results and in the abstract, but not in methods. Please also specify whether the content analysis was quantitative or qualitative. It seems from the numbers presented in the results and App 1 (utterances, beliefs, etc) that it was mainly quantitative. Usually, in qualitative research, the richness of the data is more important than the quantity of statements or of participants who contributed to a finding. Interestingly, the guide you have used (Ref 54) states: ”Frequency count of belief statements is not warranted in the case of focus groups”.

*Ethics*

-was consent written or oral? Was it preceded by information about the study and its objectives?

-Please remove ”the” before ”ethical approval”.

**Results**

-Please state the number of eligble participants who received the survey at baseline and response rate.

-Please add ”The” before ”majority”

-P. 14, line 264. The words ”more specifically, the posterior probability was” could be replaced by a semicolon after constructs. (improves conciseness)

-P-16, line 305. Focus group discussions are typically just that – discussions rather than interviews. Please consider avoiding terms such as interviewed, interviewee.

-P. 16, line 301-311. How did you determine which groups the participants belonged to?

-Please consider expanding the qualitative findings section to summarize the key findings, including the most important individual and organizational factors that was perceived to influence their EBP use. There are several findings discussed in the Discussion section (e.g. own and peers’ experience, clients’ safety and preferences, positive patient outcomes, positive attitudes, organizational barriers, etc) that are not presented in the Results section.

-Please consider clarifying ”constructs” by adding e.g. EBP or EBP-related before the word construct – in text as well as table heading and figure legend.

**Discussion**

-Please begin the discussion by summarizing key findings from the quant and qual data, including the main individual and organizational factors that influence EBP use (i.e., your research question). More or less like you have done in the conclusion, line 439-444.
A statement in line with: ”The findings indicate that x, y and z are important factors…” would be an appropriate way to summarize what you found in answer to your second research question.

-P. 17, line 327. Several of the references 56-60 are not studies of ATs and PTs so should not be used to support a statement that OTs and PTs highly value personal practice experiences….

-As a continuation of the point above, I do miss a comparison of findings among ATs and PTs with other healthcare professions such as nurses and physicians. Much EBP research has been done also among those professions.

-P. 16, line 323. Please change wording to ”EBP evolves among” instead of ”…in”.

-P. 16, line 327-329. The finding that practitioners value personal practice experience and that of their peers – I can’t see this finding reported in the Results section.

-P. 17, line 339-341. Is this a finding from your study or from previous literature? If the former, I don’t see it reported in the Results section, and if the latter, it needs a reference.

-Please discuss the gender distribution in both the survey data (83%-91%) and the focus group data (87%).

-Please discuss limitations and strengths of the study.

-Please briefly discuss generalizability and transferability of the findings, as well as reliability, credibility and trustworthiness.

**Conclusion**

-P. 21, line 440. I believe ”positive” should precede ”attitudes toward EBP”.

**References**

-Please double check ref 41. I believe there is an ”In” missing to clarify that it’s a book chapter, and the book title is abbreviated so that it seems like a journal title.
